# COVID-19 Impact on SDGs and the Fiscal Measures: Case of Indonesia

**DOI:** 10.3390/ijerph18062911

**Published:** 2021-03-12

**Authors:** Yulida Safitri, Reni Diah Ningsih, Dwi Putri Agustianingsih, Vibhas Sukhwani, Akiko Kato, Rajib Shaw

**Affiliations:** Graduate School of Media and Governance, Keio University, 5322 Endo, Fujisawa, Kanagawa Prefecture 252-0882, Japan; yulida89@sfc.keio.ac.jp (Y.S.); renidiah@sfc.keio.ac.jp (R.D.N.); agustia@sfc.keio.ac.jp (D.P.A.); vibhas@sfc.keio.ac.jp (V.S.); katoakiko1012@gmail.com (A.K.)

**Keywords:** COVID-19, sustainable development goals, fiscal stimulus, Indonesia

## Abstract

The implications of the ongoing COVID-19 pandemic have stretched far beyond human health and wellbeing, causing serious setbacks for the achievement of the Sustainable Development Goals (SDGs). Although governments worldwide have implemented different fiscal stimulus measures to mitigate the implications of COVID-19, it is important to develop a precise understanding of their focus areas to ensure if the progress of SDGs is on track. For a specific case of Indonesia, this study establishes a thorough understanding of the COVID-19 implications on SDGs, and its fiscal stimulus package through a literature review and semi-formal interviews with the core stakeholders in Indonesia. The study results highlighted that COVID-19 has varyingly affected the progress of all SDGs in Indonesia. Amongst the four pillars of sustainable development in Indonesia, the SDGs on the social and economic development pillars are stated to be the most impacted. As for the fiscal stimulus, it is perceived that it can help maintain the SDGs’ attainment progress to a certain extent, although there are several concerns on its implementation. Deriving lessons from the conducted research, the study puts forward key suggestions for the effective implementation of SDGs in the post-COVID-19 era.

## 1. Introduction

The first cases of coronavirus disease 2019 (COVID-19), a respiratory illness caused by the novel form of coronavirus called severe acute respiratory syndrome coronavirus 2 (SARS-CoV-2), were reported in China towards the end of 2019. It was later declared a pandemic by the World Health Organization (WHO) on 11 March 2020, after the virus spread to more than 110 countries and there were 118,000 confirmed cases worldwide [1]. By 5 March 2021, the global confirmed cases for COVID-19 had surpassed the staggering figure of 114 million, with over 2.5 million deaths [2]. 

Despite the fact that the COVID-19 pandemic is still unfolding, countries all over the world have distinctly executed a range of countermeasures to control and manage the spread of the virus, including the closure of educational, commercial, sports, and religious institutions [3]. In that manner, the impact of the COVID-19 pandemic has extended far beyond the global health sector, affecting the social and economic sectors as well. Border controls have been strengthened, as have lockdowns and travel bans, resulting in widespread economic closures and job losses. According to the WHO [4], over 10 million people are at risk of falling into severe poverty, and malnutrition is predicted to increase by up to 130 million people. Furthermore, the COVID-19 pandemic has also posed major challenges for achieving sustainable development in the context of increasing food insecurity, hunger, and unemployment. 

Initially, 2020 was supposed to be the marking point of the Decade of Action to keep track on the 2030 Sustainable Development Agenda, and the 17 defined Sustainable Development Goals (SDGs) [5]. Having been already behind, the progress of SDG attainment worldwide has been further pushed back due to the emergency of the COVID-19 pandemic. Specifically, in the developing nations of South-East Asia—although all the member states of the Association of South East Asian Nations (ASEAN) have geared towards achieving SDGs by 2030—none of the 17 SDGs have been met [6]. It is projected that the COVID-19 pandemic will further delay the progress of SDGs in the region [7]. 

Indonesia—like all other nations—is also struggling through the pandemic’s emergency consequences. Early in March 2020, the first confirmed case in Indonesia was recorded, and COVID-19 was then declared a national non-natural disaster by the Indonesian government on 13 April 2020 [8]. By 5 March 2021, there were more than 1.36 million confirmed cases, and 26,897 deaths [9]. The Indonesian government’s numerous countermeasures, such as large-scale social restriction, physical distancing, massive rapid testing, and work and study from home policies, have all resulted in considerable economic downturn [10]. To this extent, COVID-19’s impacts have hampered Indonesia’s progress toward the SDGs [11,12].

Referring to the Sustainable Development Report in 2020 [13], some SDGs are on track and moderately improving, while other SDGs are posing major challenges and showing a stagnant progress pattern. In the backdrop of COVID-19, Indonesia faces greater challenges in achieving the SDGs by 2030, as the economic downturn has affected households, Small and Medium Enterprises (SMEs), corporations, and the financial sector [14]. As a result, the Indonesian government implemented the fiscal stimulus to offset the multisector effects of the COVID-19 pandemic [11,14].

The fiscal stimulus and/or state budget instruments are stated to be driving the economic improvements in handling the COVID-19 pandemic and the national economic recovery program [15]. Although Indonesia’s economy grew in the third quarter (Q3) of 2020 [16], there is no clear information as to what extent the fiscal stimulus is intended for achieving SDGs during the COVID-19 outbreak, although they have been defined as critical [17]. As such, the implications of COVID-19 for SDGs’ progress in Indonesia are yet to be understood, let alone the role of fiscal measures towards the SDGs’ implementation. 

While extensive research studies have been conducted so far to study the implications of the COVID-19 pandemic, there have been very few empirical studies seeking to understand their consequences on the progress of SDGs and the relative role of fiscal stimulus measures. Furthermore, since COVID-19 economic recovery packages must be designed to not only mitigate the pandemic’s short-term repercussions, but also to “build back better” towards a more sustainable and resilient future [18,19], it becomes more imperative to understand how the fiscal stimulus initiatives have addressed the SDGs’ attainment so far.

With an aim to bridge this gap, a qualitative research design (combination of a literature review and semi-formal interviews with key stakeholders) has been adopted in this study to methodically understand the impacts of COVID-19 on the progress of SDGs in Indonesia, as well as the role of fiscal measures for keeping SDGs on track. The three key objectives of this study are: (1) to establish a wider understanding about the impacts of the COVID-19 pandemic on the progress of SDGs in Indonesia; (2) to study the stakeholders’ perception about the impact of the COVID-19 pandemic and the impact of stimulus measures towards the SDGs’ progress; and (3) to suggest feasible directions for enhancing and strengthening the implementation of fiscal measures and SDGs in Indonesia. In doing so, the study addresses two key research questions: (1) How has the COVID-19 pandemic impacted the progress of SDGs? (2) What are the perceived direct and indirect impacts of fiscal policies in Indonesia towards the progress of SDGs’ implementation? 

For the study context, it is important to highlight that the “direct impacts” refer to the immediate impacts of the fiscal measures on the SDGs, while the “indirect impacts” refer to the relatively predictable effects of the stimulus on the SDGs that could arise later in time or in the cause-and-effect relationship chain.

Overall, this paper is divided into seven sections, the first of which is the Introduction (Section 1). Section 2 provides an overview of the current scientific literature on the SDGs, as well as the implications of the COVID-19 pandemic on SDGs worldwide, and the fiscal measures. The third section introduces the case study area of Indonesia, and Section 4 explains the research methods adopted in this study. In Section 5, the research findings and analysis are presented. The following section (Section 6) provides the relevant policy recommendations to resolve the immediate issues identified through this study. The final section (Section 7) outlines the key findings, contributions, and constraints of this research.

## 2. Literature Review

This section is mainly intended to establish a foundational view of the study’s topic, based on the review of state-of-the-art scientific literature. It is divided into three sub-sections. The first sub-section provides an overview of the SDGs and their global progress. The second sub-section highlights the implications of the ongoing COVID-19 pandemic towards SDGs. The final sub-section discusses the fiscal stimulus programs implemented by governments around the world to overcome the repercussions of the COVID-19 pandemic.

### 2.1. Importance of the Sustainable Development Goals

On 25 September 2015, the 2030 Development Agenda (titled “Transforming our world: the 2030 Agenda for Sustainable Development”) was adopted by 193 countries of the United Nations (UN) General Assembly, which serves as a framework for achieving peace and prosperity around the world. As the continuation of the Millennium Development Goals (MDGs), the 2030 Development Agenda outlines 17 SDGs and 169 associated targets, which frame the integrated plan of action in a global partnership for people, the planet, and prosperity [18,20,21]. The SDGs are primarily designed to provide policy guidance and to evaluate government performance in key areas that affect the people’s and the planet’s well-being [22].

Having been implemented for five years, the global progress on achieving the SDGs appears to be lagging behind in fulfilling the targets [5,23,24]. Barbier [25] stated that while some goals have made strides, they have come at the detriment of other goals. Poverty eradication, child and maternal health improvement, school and drop-out reduction, and women’s participation are among the goals that have made some progress. However, the natural environment is still in grave danger, and inequality remains profound [5]. Now, the attainment of the 2030 Agenda for Sustainable Development faces unprecedented challenges due to the COVID-19 global pandemic [5,26].

### 2.2. Implications of COVID-19 on SDGs Worldwide

The progress of SDGs worldwide was already behind the target, and the COVID-19 pandemic brings tremendous challenges in realizing all the goals [23,27]. The pandemic has not only overshadowed the focus on SDGs, but it has also overturned the years of progress made so far [13,28]. COVID-19’s effect on SDG 1 (No Poverty), SDG 2 (Zero Hunger), SDG 3 (Good Health and Wellbeing), SDG 4 (Quality Education), SDG 8 (Decent Work and Economic Growth), and SDG 17 (Partnership for the Goals) has been the subject of extensive discussion [14,23,27,29,30,31,32]. However, the pandemic’s impacts on other SDGs have not received considerable coverage yet. 

**Figure 1:**
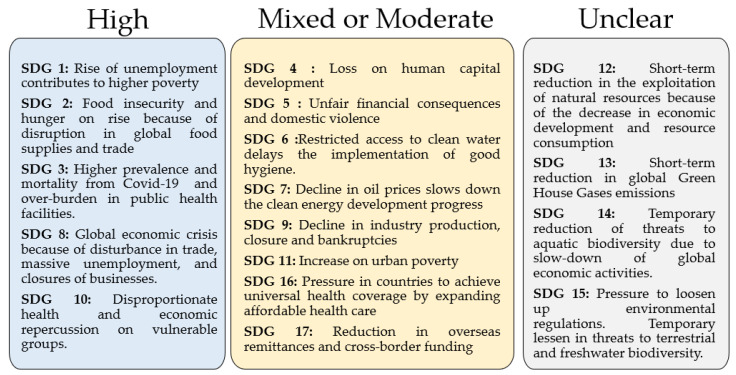
Short-term impacts of COVID-19 on the Sustainable Development Goals (SDGs) (Modified from Sachs [13]).

Based on the Sustainable Development Report in 2020 [13], the short-term impacts of COVID-19 on SDGs have been classified into three levels: High, Mixed or Moderate, and Unclear (refer to Figure 1). The “high level” category indicates that COVID-19 has had a major negative effect on the achievement of the SDGs, in which they become the key focus on SDG progress worldwide. The “mixed” or “mild” level comprise the SDGs that are experiencing moderately negative impacts through the pandemic, or both positive and negative impact on the progress of SDGs. The “unclear level” indicates that the impact of COVID-19 on the SDGs’ progress is yet to be observed.

### 2.3. Fiscal Stimulus Measures around the World

Governments all over the world implemented fiscal stimulus initiatives to counteract multi-sector effects of the COVID-19 pandemic [33]. Herein, the fiscal policies primarily refer to the changes in government expenditures and taxation structures to affect a nation’s economic conditions, and the fiscal stimulus specifically refers to the increase in government spending and tax cuts to stimulate public demand for goods and services [34]. Shortly after COVID-19 was declared a pandemic, the Governments of Japan, the United States (US), Canada, India, and South Africa all initiated massive fiscal stimulus programs, far exceeding the amount of stimulus implemented during the 2008 financial crisis. Likewise, Western European countries have already set aside up to four trillion USD for fiscal stimulus measures [35]. 

It is evident that the components of fiscal stimulus (to mitigate the impacts of COVID-19) differed from country to country. The ASEAN member states’ fiscal stimulus packages were designed to assist people, households, and businesses in all sizes to survive the impacts of the pandemic and to improve the healthcare system [36]. Other nations such as Canada, Australia, and Japan used wage subsidies to distribute stimulus to workers and employers. France has increased its fiscal stimulus package, which includes funding for research and training. Germany, South Korea, and Japan have all initiated fiscal stimulus programs aimed at green growth and digital infrastructure [37]. 

The International Monetary Fund (IMF) [37] stressed that the fiscal stimulus (public investment) is essential to strengthen economic resilience, promote long-term economic development, and to support SDGs. As such, the stimulus packages with the primary objective of improving public health infrastructure have the ability to build resilience in addition to promoting the goal of SDG 3 (Good Health and Wellbeing) [38]. Furthermore, the social protection measures may contribute to addressing the pandemic’s immediate issues while also building on long-term SDG commitments [39]. According to Zhang [40], the scope of social security policies has both direct and indirect effects in terms of preventing individuals from losing their jobs and properties, as well as indirect effects in terms of providing opportunities for the vulnerable to rise out of poverty, resulting in a more prosperous and resilient economy. 

## 3. Case Study Area—Indonesia

**Figure 2:**
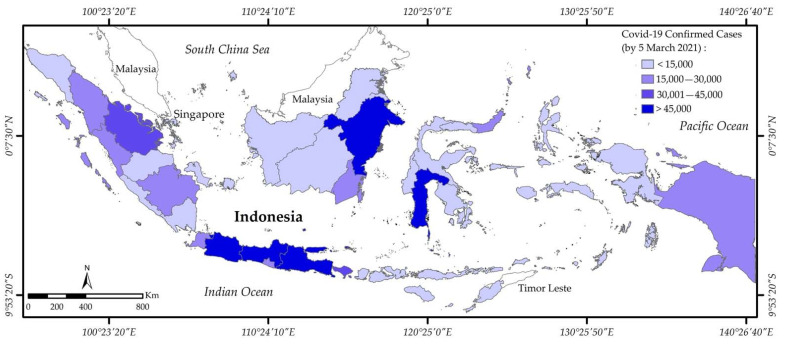
Indonesia’s confirmed COVID-19 cases by province (Image source: author).

Indonesia is one of the world’s largest archipelagic countries with over 17,000 islands. It is located south of Malaysia and the Philippines, between the Asian and Australian continents, and between the Indian and Pacific oceans [41]. The central government, regional governments (34 provinces), regencies and towns (514 cities and regencies), districts (7071 districts), and villages (81,936 villages) make up Indonesia’s governmental structure [42,43]. Regencies and cities as the sub-provincial level or local governments have greater responsibilities of management affairs and regional interests [44]. The current population of Indonesia is estimated to be 269 million [45]. More than half of the population reside on the island of Java, which has the highest rate of COVID-19 cases in the country (refer to Figure 2).

Like all nations worldwide, Indonesia has faced the widespread repercussions of COVID-19 not only in its public health but also in its economy. The COVID-19 pandemic has slowed the economic growth in Indonesia, as observed from the country’s Gross Domestic Product (GDP) growth contracting in the first and second quarters of 2020 [10,46]. In 2021, the economy of Indonesia is expected to grow at a rate of 4.9–5.1% [47]. More notably, the economic recovery after COVID-19 in Indonesia is projected to be faster due to domestic demand-oriented economic growth that makes Indonesia less vulnerable to global economic downturns [47].

For the effective implementation of SDGs, Indonesia has taken considerable efforts to integrate the goals into its development strategies and policies, and to assure that these goals are implemented at the ground level. As a form of commitment to SDG implementation, the President of Indonesia signed the Presidential Regulation (Perpres) Number 59 Year 2017 (on July 4) on Achieving Sustainable Development Goals (SDGs) [48]. The decree regulates the structure of the National Coordination Committee, the engagement of representatives of the implementation team, and the task force of government agencies and non-government agencies, as well as the roles and responsibilities of the stakeholders. The regulation also assigned 17 goals and 169 indicators, aligning them with Indonesia’s Long-Term National Development Plan (RPJPN) and Medium-Term National Development Plan (RPJMN). To support the SDGs’ attainments, the Indonesian government has also developed a number of additional documents including the SDGs Road Map, National Action Plan (RAN) and Regional Action Plan (RAD), and SDGs metadata indicators which serve as guidelines for SDG implementation [48]. 

**Figure 3:**
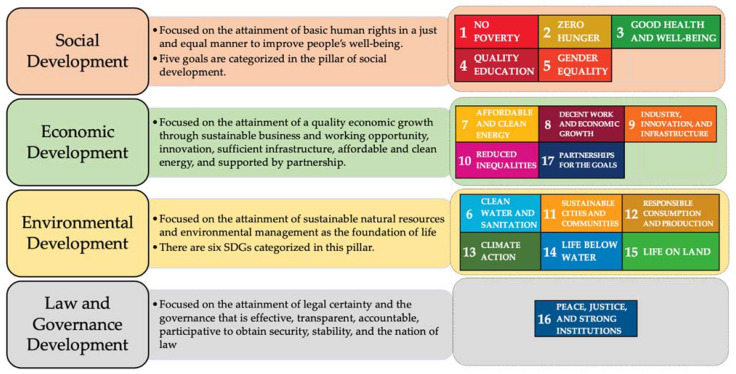
Four Sustainable Development Pillars in Indonesia (Modified from BAPPENAS [49]).

As the coordinator of SDG implementation in Indonesia, the Ministry of National Development Planning (BAPPENAS) has categorized the 17 SDGs into four development pillars [49], which are illustrated in Figure 3.

While the COVID-19 pandemic has caused significant disruptions to the progress of the 2030 Agenda, the deceleration is apparently concentrated in the social and economic development pillars of SDGs in Indonesia. The virus has taken a huge toll on public health services around the nation, and education has shifted from traditional classrooms to online platforms. As the poverty rate is expected to increase, there are also growing concerns about food insecurity. The pandemic is also likely to curtail the progress in achieving gender equality [11,49]. 

Under the cornerstone of economic development, the success of the SDGs has had significant ramifications. As explained earlier, Indonesia’s economic growth has plummeted after the onset of COVID-19, and so has inequality. The advancement of clean and affordable energy has slipped down the priority list [32]. Reportedly, the environmental development pillar has experienced a positive impact of the pandemic, although it is more likely to be temporary [50]. Furthermore, the effects of COVID-19 on the law and governance development pillar have not yet been discussed widely.

**Figure 4:**
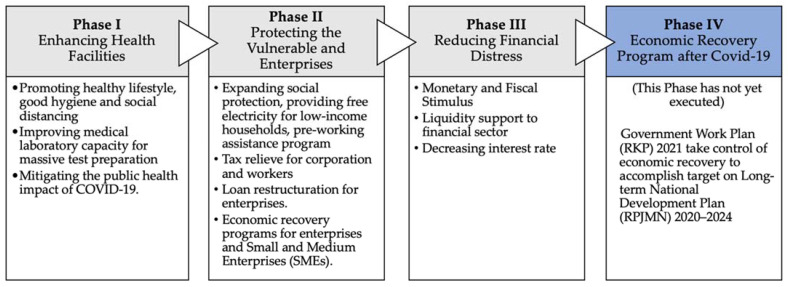
The timeline of COVID-19 mitigation policies (Modified from BAPPENAS [11]).

Altogether, the COVID-19 pandemic has had a wide-ranging effect on the people as well as the economy in Indonesia. To overcome the economic meltdown, the Indonesian government has taken several efforts and established fiscal policy measures under the National Economic Recovery (PEN) program. The economic recovery package of Indonesia (PEN) comprised of around 4.2% of the nation’s GDP, and its implementation is being performed in four phases [11], as illustrated in Figure 4. Phases 1 to 3 are currently ongoing and will continue until the COVID-19 pandemic is handled. The main focus of these phases is to implement fiscal stimulus measures that are delivered directly to a variety of target groups, including individuals, paramedics, corporations, SMEs, etc. The main aim is to boost Indonesia’s economic growth out of recession. Phase 4 has not yet been implemented, and it will be executed only after the COVID-19 pandemic has been completely handled. The dedicated amounts of fiscal stimulus in Indonesia are shown in Table 1.

In order to fund the COVID-19 mitigation and the PEN, the Indonesian President has issued the Indonesian President Instruction (Inpres) No. 4 year 2020 on government programs’ refocusing, budget reallocating, and procurement of goods and services to accelerate COVID-19 mitigation [52]. The Inpres instructed all ministries, institutions, provincial governments, and local governments to reallocate their annual expenditures for COVID-19 mitigation and PEN [52,53]. Within Indonesian PEN, the largest amount of fiscal stimulus is allocated towards the social protection category, followed by SMEs, business incentives, sectoral and regional government, health, and corporate financing.

## 4. Research Methods

To study the impact of COVID-19 on SDGs in Indonesia, two steps of research were conducted in this study. Firstly, a methodical literature review was conducted to establish a basic understanding of the research subject. Relevant documents were identified from the selected research databases of Scopus, Science Direct, ResearchGate, and Springer by executing search queries for keywords such as “COVID-19”, “SDGs”, “COVID-19 fiscal stimulus” and “Indonesia”. While the research documents related to COVID-19 and SDGs are still very few, a total of 83 research documents were identified (as of December 2020) through keyword search queries in all defined research databases. These documents were then sorted by their title and abstract to meet the relevance for this study, after which 16 of these documents were taken into consideration. In addition, an online search was also conducted to find relevant policies, official reports, and statistics from the government, international organizations, (Non-Governmental Organizations) NGOs, media releases, and academic research. 

In the second step, semi-formal interviews (through online meeting platforms) were conducted with key stakeholders in Indonesia to substantiate the literature findings. The online mode was explicitly chosen with respect to the core need to maintain social distancing and to avoid any unintended consequences of this study. The key stakeholders were identified in consideration of their roles and responsibilities in SDG implementation and fiscal stimulus policy in Indonesia, which are mostly conducted by Indonesian government agencies. Thus, representatives from three different Indonesian government agencies were approached to be interviewed in this research: (1) SDGs Secretariat of BAPPENAS: the coordinating agency for SDGs Implementation in Indonesia; (2) Ministry of Finance: the agency that is responsible for fiscal policy; (3) Statistics Indonesia: the SDGs database coordinating agency. To further widen the range of stakeholders interviewed in this study, academic experts, NGOs, community groups, and the private sector were also identified based on their work on the field of SDG implementation in Indonesia. 

During November–December 2020, a total of 11 interviews were conducted with a range of stakeholders from Indonesia as follows: one expert form the SDGs Secretariat of BAPPENAS, two experts from the Ministry of Finance Indonesia, two experts from Statistics Indonesia, one expert from SDGs Research Centre, two academic experts, one expert from an NGO, one expert from a community group, and one expert from the private sector.

Three broad research topics were prepared for guiding these interviews: (1) The impact of COVID-19 on SDG attainment in Indonesia; (2) The role of fiscal stimulus to keep SDG progress on track; (3) The impact of fiscal stimulus on SDG progress in Indonesia. Based on these topics, the authors prepared specific research questions to initiate the discussions during the semi-formal interviews as follows: (1) Describe the impact of the COVID-19 pandemic on SDG progress in Indonesia; (2) Which SDGs are the most affected by the pandemic?; (3) Do you think the fiscal stimulus will help to keep SDG progress on track?; (4) Which SDGs will be directly impacted by the fiscal stimulus?; (5) Which SDGs will be indirectly impacted by the fiscal stimulus? 

The average duration for each of the interviews was around 60 min and all the interviews were documented. In order to effectively keep track of all interview responses, the authors also prepared matrices to list down the direct and indirect impacts on all 17 SDGs and the fiscal stimulus. These matrices were later brought to Microsoft Excel for analysis and data display. Based on the responses from the interviews, a deeper understanding regarding the impact of the COVID-19 pandemic on SDG implementation in Indonesia and the impact of fiscal stimulus towards SDG progress was established, and the literature findings were accordingly substantiated.

## 5. Results

### 5.1. Impacts of COVID-19 on SDGs in Indonesia

Based on the insights gained through the literature research and semi-formal interviews, it has been inferred that the progress of SDGs’ realization in Indonesia was lagging even before the pandemic. The interviewees even raised specific concerns on the progress of SDG attainment in Indonesia before COVID-19, which are explained as follows:

1. Insufficient SDG knowledge dissemination

As a new development concept adopted from an international agreement, SDG knowledge was first disseminated at the national government level, from where the knowledge was transferred to provincial and city/regional governments. However, some stakeholders argued that the knowledge transfer had not been well implemented. A familiarity gap persists between the national government and other tiers of governments below it. The knowledge on SDGs and their implementation is primarily possessed by those in the national government, whereas the other government levels have inadequate knowledge of SDGs, let alone the ability to translate them into their development programs.

2. SDGs are not yet mainstreamed into the development programs in provincial and local government levels

Although it has been instructed by the national government of Indonesia to main-stream SDGs into development programs in all government levels, some stakeholders claimed that many provincial and local governments have not yet been able to do so. Through the interviews, it was discovered that certain government programs are not yet aligned with the SDGs, which hinders their progress. In addition, several programs are aligned with SDG attainment but are not yet synchronized on the programs’ report. Herein, the interviewees highlighted that the SDG roadmap set by BAPPENAS is yet to be translated into development programs by all government levels and institutions, particularly provincial and city/regional governments.

The interviewees perceived that COVID-19 would delay Indonesia’s progress towards achieving the SDGs. Further, some SDGs are perceived to be more impacted than others, especially those under the social and economic development pillars. 

**Figure 5:**
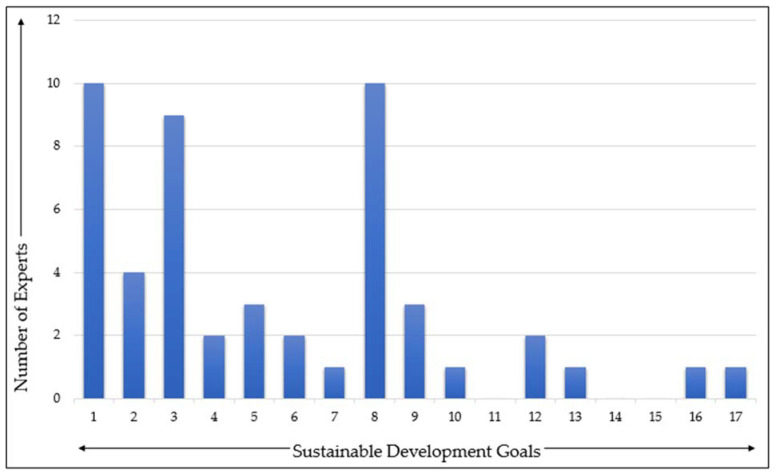
The impact of COVID-19 on SDGs identified by the stakeholders’ interviews.

**Figure 6:**
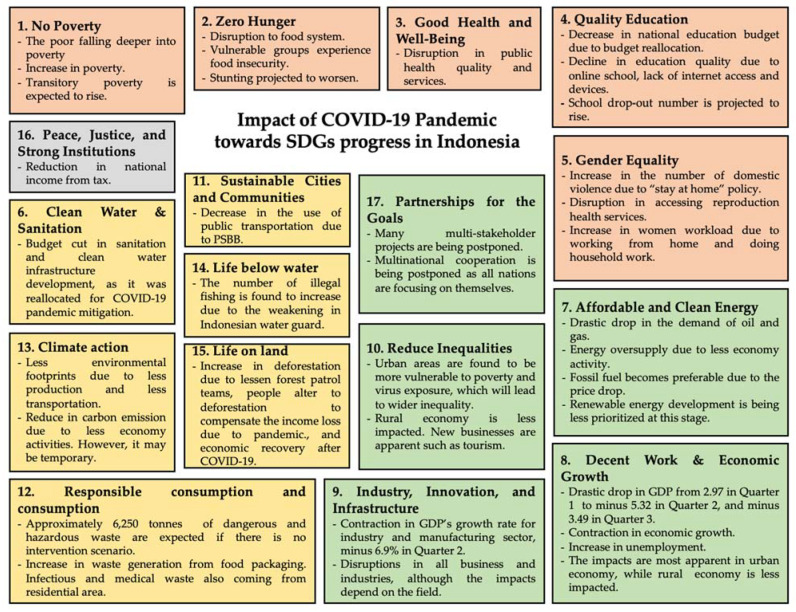
The summary of COVID-19 pandemic towards SDG progress in Indonesia.

Derived through the stakeholder perception, Figure 5 below depicts the impact of the COVID-19 pandemic on the attainment of SDGs in Indonesia. The top three most impacted goals by COVID-19, according to stakeholders, are Goal 1 (No Poverty), Goal 8 (Decent Work and Economic Growth), and Goal 3 (Good Health and Well-being), while Goal 11 (Sustainable Cities and Communities), Goal 14 (Life Below Water), and Goal 15 (Life on Land) are perceived to have experienced little impact from COVID-19. The more specific impacts of COVID-19 on different SDGs in Indonesia are discussed in the following sub-sections, which are organized in accordance with the four pillars of sustainable development (explained in Section 3) defined by BAPPENAS, the coordinator of SDG implementation in Indonesia. The overall summary of the impacts of COVID-19 pandemic is depicted in Figure 6. 

#### 5.1.1. Impacts on Social Development Pillar

In the wake of the COVID-19 pandemic, achieving SDG 1 (No Poverty) would be extremely challenging in Indonesia. The poverty rate is expected to rise as a result of large-scale social restriction policies which have been implemented by provincial, city, and regional governments all around Indonesia. Many people have lost their jobs and enterprises as a result of enforced social restrictions. The vulnerable groups of society are expected to fall into poverty, and those who are poor are expected to fall into deeper poverty. As per BAPPENAS [11], the poverty level is projected to reach 9.7–10.2% of the population. Additionally, according to the World Bank [54] survey, lower-middle-income households will face income shocks as a result of pandemic, with job losses in urban areas being more prevalent. These literature findings have also been corroborated with the stakeholder interviews, wherein it was pointed out that COVID-19 will likely exacerbate the poverty in Indonesia.

The disruption of the food system due to COVID-19 restrictions has raised concerns for SDG 2 (Zero Hunger). BAPPENAS [11] underlined that the people who experienced a reduction in income levels would see a decrease in the quality and quantity of food available, and the most disadvantaged members of the community may even experience hunger. Through the semi-formal interviews, it was reviewed that the number of stunting cases would likely worsen in the long run as a result of a decline in food nutrition. In that manner, COVID-19 may reverse the moderately growing development of SDG 2 (Zero Hunger).

The pandemic also has a major effect on SDG 3 (Good Health and Well-being) according to the stakeholders interviewed. While the disruption in public health is apparent, the public healthcare services are presently more focused on controlling the pandemic, which is likely to disrupt the whole public service. 

The reallocation of the education budget for COVID-19 mitigation measures has impacted the national education program [55], as well as constrained the attainment of SDG 4 (Quality Education). To suppress the pandemic, the government has also implemented the school from home scheme, wherein the efficacy of online learning remains to be determined in the long run. Additionally, some students have been unable to access the online classes due to remote locations, a lack of sufficient devices, book and supplies, and financial constraints for internet access [56]. Through the semi-formal interviews, the potential issue of an increase in the school dropout number also came to light, as more students are projected to leave school to support the falling family incomes.

The achievement of SDG 5 (Gender Equality) is also affected by COVID-19. Several reports have highlighted the rise in physical assault cases within the first six weeks of the large-scale social restriction in Indonesia. From March to April 2020, the number of domestic violence cases was reported to be doubled [57]. The insights obtained from the semi-formal interviews further revealed that women are at greater risk due to the declining economy because they lack decision-making capacity, their basic needs are not met, and access to health reproduction services is restricted. Furthermore, working women now have more domestic responsibilities as well as the additional burden of childcare.

#### 5.1.2. Impacts on Economic Development Pillar

SDG 7 (Affordable and Clean Energy) was perceived as hard to accomplish even before COVID-19. Since the pandemic, the progress has become much more hampered as the energy demands have plunged due to the closure of offices and shopping centers, and a drop in flight frequency. This has brought the fossil fuel price down significantly [11]. As a result, fossil fuel has once again outshone renewable energy sources [58]. Through the semi-formal interviews, it was highlighted that the research and development of clean and affordable energy will now be much less prioritized than before COVID-19, since the budget of renewable energy has been reallocated for COVID-19 mitigation.

The economic impact of the COVID-19 pandemic in Indonesia is highly prominent, making SDG 8 (Decent Work and Economic Growth) harder to attain. Indonesian economic growth contracted to minus 5.32% in the second quarter of 2020 [10]. The country has not experienced such a severe economic downturn since the 1998 Asian financial crisis [59]. Due to declining demand, the number of workers in the sub-sectors of trade, processing, transportation and warehousing, as well as accommodation, has decreased. Many workers in the tourism industry (cafes and restaurants) do not even have access to social security [11]. The economic growth contraction thus increases unemployment, especially for those in urban settings [59]. Further, through the stakeholder interviews, it was discovered that the rural area economy is less impacted because it is mainly supported by the agricultural sector. Additionally, new tourism sites have opened in rural areas that introduce COVID-19 spread prevention measures.

BAPPENAS [11] reported that the progress of SDG 9 (Industry, Innovation, and Infrastructure) has been affected by COVID-19 due to a range of its implications on various sectors, for example, the growth of the food and beverage industry has slowed down due to the fall in foreign demand. Because of the decline in tourism and air-travel, the aviation and tourism industries are also struggling to stay afloat. Aside from tourism, the Purchasing Manager Indexes (PMI) for manufacturing and the fiscal of imports to Indonesia have both been stated to be on the decline [11]. Through the semi-formal interviews, it was revealed that the impact of COVID-19 is dependent on the type of industry. Some industries, such as the tourism and manufacturing industries, experience harder impacts due to the large-scale social and mobility restriction. Other industries that involve export and import activities are disrupted by the lockdown policies worldwide which restrict the global trade.

While COVID-19 has widened the inequalities, the attainment of SDG 10 (Reduced Inequalities) has become far more challenging. Inequality has risen primarily due to the disproportionate impacts of COVID-19 on different social groups, such as on the informal sector. While the urban society has been shown to be more vulnerable to poverty, they are also at a higher risk of virus exposure due to high population density, slum living, and limited access to health services. Furthermore, the rural economy is less affected by the COVID-19 pandemic, at least in the early stages of the pandemic. Recently, the economy in rural areas has returned to normal, and in some cases has even grown because of the new opening for tourism with COVID-19 prevention measures. 

While no scientific study has discussed the implication of COVID-19 on SDG 17 (Partnerships for The Goals), it is perceived to be impacted based on the semi-formal interviews. Many multi-stakeholder initiatives have been put on hold due to COVID-19, which will hamper the progress toward this goal. Multinational collaborations on various development projects have been postponed due to the fact that all countries are presently focusing on themselves.

#### 5.1.3. Impacts on Environmental Development Pillar

SDG 6 (Clean Water and Sanitation) achievement was on track before COVID-19 [25]; however, in the semi-formal interviews, it was revealed that the sanitation and clean water infrastructure development budget has been reallocated for COVID-19 pandemic mitigation, which may slow down its progress.

SDG 11 (Sustainable Cities and Communities) is also impacted by COVID-19 in varying ways. According to BAPPENAS [24], the use of public transportation is declining due to the large-scale social restriction. While the means of public transportation have been constrained, people tend to commute more in their private vehicles to avoid crowds. 

During the pandemic, the rise in medical waste and plastic packaging has been a major concern for the environment as people use disposable packaging to prevent the spread of the virus [11]. Without an intervention scenario, approximately 6250 tons of dangerous and hazardous waste are expected to be generated [60], posing a threat to the achievement of SDG 12 (Responsible Consumption and Production).

In relation to SDG 13 (Climate Action), the pandemic reduced the commitment to Climate Action [61]. According to the semi-informal interviews, the large-scale social restrictions to mitigate COVID-19 impacts have resulted in a reduction in carbon emissions; however, after the pandemic ends, these emissions could significantly increase. While the environment is undoubtedly improving in the meantime due to reduced economic activities, BAPPENAS [11] highlighted that the air quality in many major cities in Indonesia has improved due to the large-scale social restriction. However, the stakeholder interviews perceived that this positive impact will only be temporary because post-pandemic economic recovery policies will most likely be introduced with Business-as-Usual (BAU) orientation instead of a green-economy approach.

The data availability for SDG 14 (Life Below Water) was limited even before the pandemic, and the pandemic has further exacerbated the situation, making it difficult to understand the impacts of COVID-19 on the attainment of this goal [12]. Regardless, there has been a growing concern regarding this goal’s progress during the pandemic. The number of illegal fishing incidents increased during the pandemic due to fewer patrols on Indonesian water [12,62].

For Goal 15 of Life on Land, the extent of deforestation is found to be increasing during the pandemic [63]. While the pandemic has affected many households due to job loss, deforestation has become one of the solutions to feed the family. Moreover, the patrol teams in forest areas are not fully operational due to the pandemic. Thus, illegal activities are easier to be executed [64]. What is more concerning is that once the pandemic is controlled, the economic recovery will almost certainly be accompanied by increased deforestation due to loosening regulations and patrols [65].

#### 5.1.4. Impacts on Law and Governance Development Pillar

Goal 16 (Peace, Justice, and Strong Institutions) is affected by the pandemic as the restriction on public services on court processes disrupts the stability in the legal and judicial fields [66]. Further, during the semi-informal interview, the key stakeholders disclosed that the tax revenue will likely experience a significant contraction due to the economic slowdown and stimulus provision.

Herein, the impacts of COVID-19 towards SDGs attainment in Indonesia have been presented, combining both literature review and stakeholder interviews as depicted in Figure 6 below.

### 5.2. Role of Fiscal Measures to Keep SDGs on Track

Based on the understanding obtained through the semi-formal interviews, fiscal measures under the national economic recovery of Indonesia were initiated as emergency measures to alleviate the impact of the COVID-19 pandemic. They were launched with the primarily goal of addressing the health fallout of the pandemic, while simultaneously mitigating social and economic impacts. Along the way, the Indonesian government has pledged to align the economic recovery measures with the SDG framework. The purpose was to expand the impact of fiscal stimulus measures to not only respond quickly to the repercussions of the COVID-19 pandemic but also to keep the 2030 sustainable development agenda on track [67]. Broadly, the fiscal measures are perceived to contribute to maintaining the SDGs’ progress in Indonesia in three core development pillars (explained in Table 2), and limited contribution is expected towards the pillar of law and governance development.

Although it is perceived that fiscal measures may help to keep the attainment of Indonesian SDGs on track, several concerns were highlighted by the stakeholders interviewed in regard to the implementation of fiscal measures:

1. The possibility of misconduct in stimulus implementation

The key interviewees argued that although the amount allocated for fiscal stimulus is not as large as expected, it will at least benefit vulnerable people, if well implemented without any misconduct such as corruption and fraud. However, the possibility of misconduct is rather high owing to the lack of accountability in the stimulus delivery process. Clean, transparent, and accountable tender mechanisms may be overlooked for the sake of ensuring a speedy stimulus implementation. This concern is also highlighted by The United Nations Office on Drugs and Crime (UNODC) [72], which stated that, under the Indonesian Government Regulation (Perpu) No. 1 Year 2020 on State Financial Systems for the Management of Corona Virus Disease 2019 (COVID-19) and/or Encounter the Threat to National Economy and/or Financial Stability, the government officials are exempted from civil and criminal liability during the implementation of this regulation if it is based on “goodwill and according to the law”. However, the definition and criteria of “goodwill” have yet to be established. Thus, it can open up a chance of misconduct.

2. Unsynchronized database at all government institutions

The semi-formal interviews revealed that the social protection fiscal measures may be beneficial only if they are delivered to the accurate beneficiaries. However, some interviewees highlighted that the database used for fiscal measure distribution is poorly maintained and updated. Each ministry has its own database, and so have the provincial and city/regional governments. These databases have not yet been integrated across government institutions and at all levels. As the accuracy of the beneficiary data is still inadequate, the distribution of social protection packages is often disrupted. For instance, during the ongoing COVID-19 crisis, some households received the social protection packages despite not meeting the eligibility criteria, while there have been cases where qualifying recipients were overlooked.

3. Lack of resources and capacity in fiscal measures’ implementation

A large number of fiscal measures are distributed by the local governments in Indonesia. However, some interviewees argued that the local governments have not yet obtained sufficient capacity in managing a huge amount of emergency budget which must be distributed in a short period of time. This could lead to further implementation problems such as delayed delivery and a chaotic distribution process. The new work-from-home setting caused by COVID-19 has also hampered the fiscal measures’ implementation. The stakeholders perceived that the Indonesian government is not yet able to adopt a work-from-home setting both in terms of the manpower capacity and infrastructure.

### 5.3. Perceived Direct and Indirect Impact of Fiscal Measures on SDGs’ Progress

**Figure 7:**
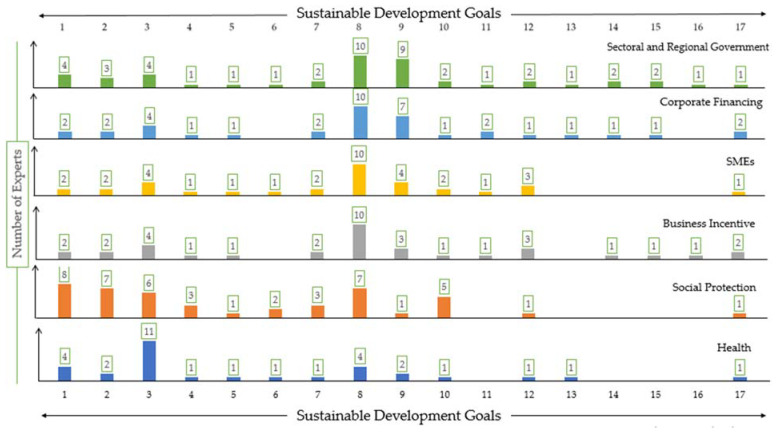
Perceived direct impact of fiscal measures on SDG progress in Indonesia.

Figure 7 illustrates the perceived direct impact of fiscal measures on SDGs’ progress in Indonesia based on the stakeholders’ semi-formal interviews. The bar illustrates the perceived direct impact of each fiscal stimulus category towards all 17 SDGs. The number in each colored bar represents the number of stakeholders (out of 11 stakeholders interviewed) who perceived the direct impact of each stimulus to corresponding SDGs.

Noticeably, the goals within the “economic” and “social development” categories are perceived to be the most directly impacted goals by the fiscal measures. However, goals under the environmental and law and governance development categories do not experience as much direct impact. Most stakeholders regarded that SDG 8 (Decent Work and Economic Growth) will be mostly impacted directly by the stimulus, followed by SDG 3 (Good Health and Well-being), and SDG 9 (Industry, Innovation, and Infrastructure), respectively. The reason is that the stimulus is largely allocated towards economic recovery, whether through SMEs financing, business incentives, sectoral and regional governments, and corporate financing.

The stimulus on the sectoral and regional government category is recognized as being able to give a direct impact to all 17 SDGs, although the amount of the budget allocation is not as high as other economy related stimulus categories. It is because the stimulus aimed for the sectoral and regional government will be spent by ministries and local governments all over Indonesia for a variety of purposes. This stimulus enables the Indonesian government in all levels to execute a broad range of programs that may cover all SDGs, unlike other stimulus categories which are specifically addressing only those within their field.

**Figure 8:**
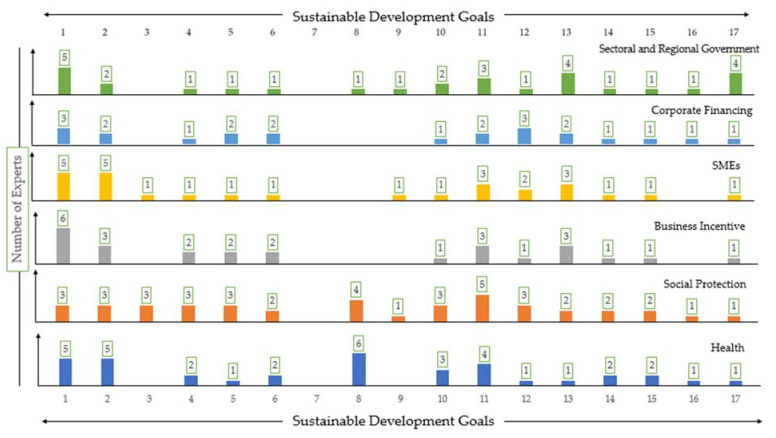
Perceived indirect impact of fiscal measures on SDG progress in Indonesia.

Figure 8 highlights the indirect impacts of fiscal measures towards SDGs obtained from the stakeholders’ interviews. Although the “social” and “economic development” pillars are perceived to remain dominant in experiencing indirect impacts of the stimulus, the goals under the “environmental development” pillar are expected to be impacted as well in the future. Notably, the stakeholders perceived that none of the fiscal measures will address SDG 7 of Affordable and Clean Energy. They argued that achieving clean and affordable energy will be even less prioritized during economic recovery. The SDGs under the environmental development pillar, SDG 6 (Clean Water and Sanitation), SDG 11 (Sustainable Cities and Communities), SDG 12 (Responsible Consumption and Production), SDG 13 (Climate Action), SDG 14 (Life Below Water), and SDG 15 (Life on Land), would thus be impacted in the future as a prolonged impact of the stimulus. 

## 6. Discussion

Evident through the research findings, the attainment of SDGs in Indonesia has been impacted by the pandemic (as also discussed in Section 5.1), although there are some gaps in comparison to the UN assessment of the short-term impacts of COVID-19 on SDGs (highlighted in Section 2.2). Based on semi-formal interviews, the stakeholders perceived that COVID-19 has a significant effect on SDG 1 (No Poverty), SDG 3 (Good Health and Well-being), and SDG 8 (Decent Work and Economic Growth). Meanwhile, based on the UN assessment, SDG 2 (Zero Hunger) and SDG 10 (Reduced Inequality) are also assessed as highly negatively impacted by COVID-19.

Further, like many other nations around the world, the Government of Indonesia has also taken fiscal measures to mitigate the multisector fallouts from the COVID-19 pandemic. The economic packages launched by the Indonesian government are aimed to support domestic consumption and prevent rapid poverty and rising unemployment. Although the fiscal measures do not explicitly seek to minimize the impacts of COVID-19 on SDGs, it can be concluded based on the semi-formal interviews that the fiscal measures would have a direct impact on SDG 8 (Decent Work and Economic Growth) and SDG 3 (Good Health and Well-being) (explained in Section 5.3), in which these goals are perceived as highly impacted by COVID-19 based on the UN assessment of COVID-19’s short term impacts on SDGs (refer to Section 2.2). 

Based on the understanding gained through the existing literature and semi-formal interviews, the authors suggest specific policy recommendations (in the following three subsections) to address the identified concerns on fiscal measures and SDG implementation in Indonesia (presented in Section 5.1).

### 6.1. Ensuring Good Governance Practice

As discussed in Section 5.2, fiscal measures for COVID-19 mitigation require the Indonesian government to administer a significant amount of money intended to benefit the people and the economy. However, the government is under pressure to complete everything within a short period of time. Executing fiscal measures in such a tight timeframe necessitates more resources and capacity, which some stakeholders perceived that the government is lacking (refer to Section 5.2). Additional havoc on government bureaucracies came from the work-from-home and mobility restrictions policy. An online setting in doing government jobs adds more challenges in orchestrating fiscal measure implementation. To compensate such shortcomings, it is important for the government to be agile in facing the crisis and to come up with innovations that are data-driven and science-based to compensate the shortcomings on resources and capacity. Moreover, good and strong coordination among government institutions and across government levels are essential to ensure the effectivity in fiscal package distribution, which is also emphasized by Antara News [73].

As highlighted in Section 5.2, there is a risk of misconduct in the implementation of emergency packages to mitigate the COVID-19 pandemic. These emergency packages may possibly have been implemented with the absence of necessary inspection procedures and mechanisms with an aim to expedite packages’ distribution to those in need. Therefore, potential fraud, misuse, and corruption are increasing [72]. As discussed in Section 5.2, the stakeholder interviews highlighted that good governance practice is required to ensure there is no leakage and fraud in the process of implementing and distributing the fiscal measures from the government to the people. Although some have argued that the amount of the stimulus is insufficient to prevent poverty from worsening, at least it can help even a little bit. Therefore, it is important to implement clean and transparent governance of the fiscal stimulus packages.

### 6.2. Integrating Beneficiaries’ Database to Improve Accuracy of Targeted Groups

The allocation of the economy packages for social protection has brought forth concerns from the key stakeholder perception (explained in Section 5.2). Some interviewees stated that the budget allocated for social protection is not sufficient to overcome the economic repercussions of the COVID-19 pandemic. Sparrow et al. [62] also highlighted that the method of distribution for the social protection stimulus was based on pre-COVID-19 schemes, such as direct fund transfer (BLT), the Hopeful Families Program (PKH), the food assistance program, and the village cash support program. These existing schemes mostly target rural areas and the poor. However, based on the insights gained from the semi-formal interviews, the pandemic hits people in urban areas harder due to reduced income and job loss (Section 5.1). It is the lower-middle-income households which are prone to economic shocks due to the pandemic. Many are likely to fall into poverty for the first time, and others may fall back into poverty. Therefore, the current schemes of social protection package distribution may not address those newly poor households.

As the schemes are organized by different ministries, the beneficiaries’ database is not integrated across ministries and each ministry has its own database (Section 5.2). This has caused overlap and outdated data in emergency package distribution. Some households who are not in poverty accepted the emergency packages, while some poor families have not been identified as beneficiaries. Thus, integrating databases across ministries and governmental levels is essential to improve the accuracy of the targeted beneficiaries in the distribution of emergency packages. The COVID-19 pandemic amplifies the importance of an innovative information system which is well integrated and can accommodate a rapid data change.

### 6.3. Mainstreaming SDGs at Different Government Levels

As the Indonesian government is committed to mainstream SDGs in its economic recovery program, it is imperative to ensure the mainstreaming of SDGs in all government institutions and across all government levels. The stakeholders interviewed in this study also highlighted that one of the key challenges of attaining SDGs in Indonesia is the gap of knowledge of the SDGs themselves existing within the government institutions, as discussed in Section 5.1. The stakeholders’ perception is also highlighted, which stated that many local governments have not yet synchronized their programs to SDGs, although they have been instructed to by the central government. The reason is that they do not yet have comprehensive knowledge about SDGs; therefore, they cannot identify whether or not their programs are aligned or could be aligned with SDGs. As sectoral and regional governments are included as the fiscal stimulus beneficiaries under PEN, it is important to allocate this stimulus to programs that are aligned with SDGs.

## 7. Conclusions

While the wide-ranging implications of COVID-19 on SDGs worldwide are yet to be fully understood, this study presented a specific overview of COVID-19 implications on SDGs for the context of Indonesia. Building over the foundation of the existing literature, semi-formal interviews were conducted with the key stakeholders in Indonesia that are directly involved in the implementation of SDGs. In addition to identifying the most affected SDGs through specifically defined interview questions, this study has also derived the perceived direct and indirect impacts of the fiscal stimulus measures on different SDGs. In the backdrop of the limited research conducted so far on the implications of COVID-19, it is hoped that this research will provide useful insights and directions to the decision makers for enhancing the implementation of SDGs in the post COVID-19 era.

The findings highlighted that COVID-19 impacts all 17 SDGs, although disproportionately. Based on the four pillars of sustainable development defined in Indonesia, the SDGs in the social and economic development pillars are seen to be most impacted by COVID-19, while the pillars of environmental and law and governance development are not as highly impacted. The semi-formal interviews also helped to identify the role of fiscal measures in maintaining SDG progress, wherein the fiscal stimulus is perceived to be able to help in maintaining the SDG progress to some extent, except in the law and governance development pillar. The direct and indirect impacts of the stimulus towards each SDG were also identified. Further, the semi-formal interviews, as the key strength of this study, have also brought forward the stakeholders’ concerns on the progress of SDG implementation and fiscal stimulus implementation.

This study contributes to the existing knowledge of COVID-19, SDGs, and fiscal stimulus by providing a precise overview of COVID-19’s impacts on SDG attainment in the specific case of Indonesia. Building over the country’s governance profile and fiscal stimulus measures against the COVID-19 pandemic, the study methodically presented the research findings based on a literature review and stakeholder interviews. Through this study, specific policy recommendations were derived, in which they were established based on the stakeholders’ concerns on the COVID-19 fiscal measure implementation. Deriving specific lessons from the case of Indonesia, the study also highlighted the core need for aligning the COVID-19 fiscal stimulus with SDG attainment in order to keep the local level progress of SDGs on track, which is now is highlighted as necessary to be done. 

Towards the end, it is important to acknowledge the following limitations of this research. The study has been conducted in reference to the existing literature on COVID-19’s impacts on Indonesia and semi-formal interviews with the key stakeholders. However, the implications of the COVID-19 pandemic for SDGs may further transform, until the pandemic is fully controlled. As such, the future scope of this study necessitates a further detailed literature analysis and stakeholder perception study. It is also important to note that this research was mainly conducted through the online mode, but there is a further need for primary surveys in the field, and analysis of the secondary data. The future scope of this study also includes the mapping of the before and after COVID-19 progress of SDGs to determine the development gaps based on both primary and secondary data, and accordingly, to work for the timely attainment of SDGs. Another possible area of future research would be to investigate the impact of the COVID-19 pandemic on the environmental related goals with more data availability.

## Figures and Tables

**Table 1:** Indonesia’s fiscal stimulus against COVID-19 pandemic (Modified from Ministry of Finance [51]).

Fiscal Stimulus	Amount (IDR)	Amount (USD)
Health	87.55 Trillion	6.28 Billion
Social Protection	203.9 Trillion	14.62 Billion
Business Incentives	120.61 Trillion	8.65 Billion
SMEs	123.46 Trillion	8.85 Billion
Corporate Financing	53.57 Trillion	3.84 Billion
Sectoral and Regional Government	106.11 Trillion	7.61 Billion
Total	695.2 Trillion	49.85 Billion

**Table 2:** Role of fiscal measures in SDG progress in Indonesia.

Goals	Role of Fiscal Measures in SDG Progress in Indonesia
Social Development Pillar	Fiscal measures act as an intervention which can prevent the poverty rate worsening. Without the social protection relief measures, The World Bank’s Indonesia COVID-19 Observatory [67] estimated that potentially 5.5 to 8 million Indonesian people may fall into poverty. In addition, government emergency packages assist the people in supplementing the reduced non-food and food expenditure [59,68]. Fiscal measures will help in ensuring food security, accessibility and food quality for the civilians and maintain the infants’ stunting rate. Fiscal measures under the health category amounting to USD 6.2 billion will contribute to ensuring the accessibility of health services for more people by widening national health insurance coverage. Fiscal measures will contribute to ensuring the quality of education by providing aid for cellular data. Fiscal measures targeting SMEs will help women to keep their business afloat.
Economic Development Pillar	Electricity subsidies will assist people to ensure that their households have access to energy during the economic downturns [69]. Pre-working scheme enables the unemployed to gain access to career training to develop their skills. They will also obtain an extra cash transfer after finishing the training. Business and corporate incentives are aimed at labor-intensive programs in order to increase multiplier effects. Although economic growth is still contracting, fiscal measures are perceived to prevent the Indonesian economy from worsening.
Environmental Development Pillar	Sectoral and regional government stimulus contribute to achieving the goal of Sustainable Cities and Communities. Some programs under the sectoral and regional category are geared toward fighting climate change. However, Hadi et al. [70] argued that the stimulus provided by the National Economic Recovery Plan of Indonesia is primarily targeted at sectors which contribute significantly to Green House Gas (GHG) emissions. The orientation of the National Economic Recovery Program (PEN) remains based on business as usual, with no long-term green economy orientation [71].
